# WORKING AFTER AGE 65: THE IMPORTANCE OF PRIOR MENTAL HEALTH–RELATED SICKNESS ABSENCE AND DISABILITY PENSION

**DOI:** 10.1093/geroni/igae098.0925

**Published:** 2024-12-31

**Authors:** Aleksiina Martikainen, Kristina Alexanderson, Pia Svedberg, Kristin Farrants

**Affiliations:** Karolinska Institutet, Stockholm, Stockholms Lan, Sweden; Karolinska Institutet, Stockholm, Stockholms Lan, Sweden; Karolinska Institutet, Stockholm, Stockholms Lan, Sweden; Karolinska Institutet, Stockholm, Stockholms Lan, Sweden

## Abstract

With rising retirement ages and higher numbers of people extending their working life in Europe, more knowledge is needed on labor market participation after the standard retirement age. In this prospective population-based cohort study, we studied all 65-69-year-olds (n=201,263) and ≥70-year-olds (n=93,751) who lived in Sweden in 2014 and had at least some income from work. Using nationwide register microdata and Cox proportional hazard regression, we examined the association of prior mental and somatic sickness absence (SA) and/or disability pension (DP) and exit from paid work after ages 65-69 and ≥70, respectively. We found a gradual decline in paid work participation during the follow-up; approximately 80% remained in paid work at least some time during follow-up in both cohorts and both sexes, while 41% worked throughout the follow-up. Mental SA/DP was less common in both cohorts (ages 65-69: 4% women, 1.7% men; ages ≥70: 0.1% women, 0.1% men) than somatic SA/DP (ages 65-69: 27.4% women, 21.8% men; ages ≥70: 3.1% women, 2.9% men). Further, mental SA/DP marginally hindered extension of working life after age 65-69 among women (HR 1.09; 95%CI 1.06-1.13) compared to women without this type of SA/DP. However, no significant association was found among men nor among women or men aged ≥70. Our findings underscore the importance of considering prior mental health factors and possible gender differences in understanding and addressing challenges related to work continuation beyond standard retirement ages.

